# New Approach for Generating Synthetic Medical Data to Predict Type 2 Diabetes

**DOI:** 10.3390/bioengineering10091031

**Published:** 2023-09-01

**Authors:** Zarnigor Tagmatova, Akmalbek Abdusalomov, Rashid Nasimov, Nigorakhon Nasimova, Ali Hikmet Dogru, Young-Im Cho

**Affiliations:** 1 Department of Computer Engineering, Gachon University, Sujeong-Gu, Seongnam-Si 461-701, Republic of Korea; 2 Department of Artificial Intelligence, Tashkent State University of Economics, Tashkent 100066, Uzbekistan; 3 Department of Computer Science, University of Texas at San Antonio, San Antonio, TX 78249-0667, USA; alihikmet.dogru@utsa.edu

**Keywords:** synthetic medical data, type 2 diabetes, prediction of diseases, shuffling

## Abstract

The lack of medical databases is currently the main barrier to the development of artificial intelligence-based algorithms in medicine. This issue can be partially resolved by developing a reliable high-quality synthetic database. In this study, an easy and reliable method for developing a synthetic medical database based only on statistical data is proposed. This method changes the primary database developed based on statistical data using a special shuffle algorithm to achieve a satisfactory result and evaluates the resulting dataset using a neural network. Using the proposed method, a database was developed to predict the risk of developing type 2 diabetes 5 years in advance. This dataset consisted of data from 172,290 patients. The prediction accuracy reached 94.45% during neural network training of the dataset.

## 1. Introduction

Currently, AI algorithms are widely used in medicine to solve many problems, such as classification, disease prediction, risk evaluation, medical image segmentation, and image detection. However, most AI algorithms, particularly deep learning (DL) methods, require considerable data. The size of the dataset plays a crucial role in increasing the accuracy of the algorithms. The highest-performance milestone algorithm relies on a large database. Moreover, there is also a large open-access database, such as ImageNet, containing more than 1.5 million images, and the Open Image Dataset includes more than 9 million data points. However, in the medical field, although there are millions of data collected from various major hospitals around the world, there are still very few databases open to the public. As medical data include the personal information of patients, they cannot be shared for maintaining privacy. However, certain programmers are allowed to use them, keeping the identity of the patients confidential.

In existing algorithms, the number of recorded patients is either not satisfactory for training DL algorithms or not labeled. For example, the Pima Indian Diabetes Database, which is the most widely used program for training diabetes detection algorithms, contains data from only 768 patients (females). Annual survey: The Behavioral Risk Factor Surveillance System (BRFSS) of the US consists of more than 100,000 annual patient records, but the data are incomplete. Furthermore, finding an appropriate database for training AI models is difficult. In particular, it is almost impossible to find a database required to train predictive algorithms because the data collection process is time consuming and difficult. For example, there is hardly any open-access database that can be used to predict diabetes or its potential complications 5–6 years ahead. Therefore, over the past decade, considerable research has been conducted on the development of synthetic medical data. There are many reviews of these works [1,2,3,4,5]. 

Typically, there are three types of synthetic data: fully synthetic, semisynthetic, and hybrid. To develop a semi-synthetic database, the main features and statistical distribution of the specific dataset were imitated, that is, a new database that preserves the statistical distribution of the real one was developed. The main purpose of this method is to hide patient data from real databases and thus make the data close to that in the public database open to everyone without compromising privacy. The second method is a hybrid method, in which a large database is developed using a specific small dataset. The goal of such methods is to synthetically increase the amount of data in a small dataset via data augmentation. The third method involves developing a completely new dataset without using a real database. This method is typically used in cases where a database is unavailable in a particular field.

While studying the literature, it is clear that most studies have been conducted in the first and second directions. Only a few methods have been developed in the third research area, most of which are highly complex. In addition, most of these studies were aimed at developing electronic health records, and almost no research has been conducted on predicting the disease in advance. 

Furthermore, the traditional approach to generating fully synthetic medical data involved manual input from medical professionals, who had to painstakingly extract the necessary rules from the guidelines and books to create datasets. This process was not only time consuming but also prone to errors and inconsistencies. Additionally, it relied heavily on the availability and cooperation of medical personnel, which further hindered its efficiency. To overcome these challenges, our proposed method utilizes neural network algorithms and shuffling techniques. By leveraging these technologies, we can automate the process of generating synthetic medical data with minimal human intervention. This not only saves time but also ensures accuracy and consistency in the generated datasets.

Moreover, our method allows for scalability, making it suitable for large-scale studies that require extensive amounts of data. It also enables researchers to easily customize the generated datasets according to their specific research requirements. This method also addresses privacy concerns associated with sensitive medical data. Since the database is created using statistical information rather than individual patient records, it ensures anonymity while still providing valuable insights into disease identification.

The main concept of this method is as follows. Many types of statistical medical data are currently available. Most of them are open to use, and much of their data are detailed in special reports or in research papers. In the first stage, a primary dataset was developed using these statistics. Subsequently, a neural network (NN) was trained using this dataset. After every five training epochs, the data were shuffled using a special shuffling operation. The developed dataset was saved after each shuffle. The dataset for which NN showed the highest accuracy was selected. From the calculations, it was estimated that the resulting dataset obtained by this method was satisfactory for practical use. The database created using our method can be a valuable tool in training various AI algorithms for identifying and predicting type 2 diabetes 5 years in advance. Today, such database does not exist or is not open for use, but only statistical data are available.

## 2. Related Works

Medical data can be in the form of images, numbers, texts, or comments. In addition, they can be single data or time-series data. Therefore, the methods proposed for generating synthetic medical data differ. For example, generational neural diffusion models, variation autoencoders, and a generative adversarial network (GAN) are mainly used to develop synthetic medical images/data [6,7,8], whereas algorithms such as Bayesian networks [9] and classification and regression trees [10,11] are used to develop numerical (quantitative) and non-numerical (qualitative), and recurrent deep learning models are used to build time-series databases [12]. Because this study aims to develop a numerical database, the issues of generating images and developing non-numerical databases are beyond the scope of this study. Further information can be found in [13].

As we examine methods for generating numerical data, it becomes clear that the majority of the works that have been proposed recently are based on real databases. Their main purpose is to increase the security of the real database, hide the patients’ personal data, and, more precisely, change the data to such an extent that the patient’s identity cannot be recognized; this resembles high-level encryption. Synthetic databases that properly depict the original data distribution, for instance, would significantly minimize patient privacy concerns and may be freely shared in place of the original patient data. To develop a differentially private synthetic database, the authors of [14] presented deep learning algorithms that can capture the relationships between various variables. Ref. [15] has developed a high-fidelity open generator that generates synthetic data using a probabilistic relational model. This generator met certain privacy requirements and produced an imitation of the large French insured patients (SNDS) database. The most common method for generating synthetic data based on real data is to use GAN. Various modifications of GANs have been used to generate synthetic medical data, including HC_GAN, medWGAN, AC_GAN, MC_medGAN, EMR_WGAN [16], and EEG_GAN [17]. A synthetic replica of numerous databases was produced as a result of the studies described above and made accessible for public use, such as the NIH National COVID Cohort Collaborative (N3C), the CMS Data Entrepreneur’s Synthetic Public Use files, and synthetic variants of the French public health system claims and hospital dataset (SNDS) [18,19,20].

However, little research has been conducted to construct fully synthetic data. Rubin was the first to propose a method to develop a fully synthetic database [21]. Ragunathan et al. (2003) proposed methods based on combining point and variance estimates from multiple synthetic datasets that were closely related but slightly different from the combining rule for multiple nonresponse imputations [22]. Later, Drechsler et al. developed an improved version of this method to overcome its shortcomings [23]. Walonski developed a method for replicating health records using statistical data and medical records [24]. A statistically valid random shuffle method was developed to increase the cardinality of the heart failure dataset [25]. Although this is not a fully synthetic method, it is likely to be an image-augmentation method. Most of the aforementioned methods for developing a full synthetic dataset are complex; therefore, we propose a relatively easy method in this study.

## 3. Methodology

When we searched for studies on diabetes prediction or risk factor assessment, we found many statistical studies. Most of these authors have conducted large surveys to assess the risk of diabetes 5–10 years in advance to study the factors that lead to diabetes. For example, in [26], 260,000 people; in [27], 63,000 people; and in [28], more than 93,000 people were surveyed/observed over 5 to 10 years. The most important aspect of these studies is the relationship between risk factors and their possibility of causing the disease is written down in the smallest detail.

**Figure 1:**
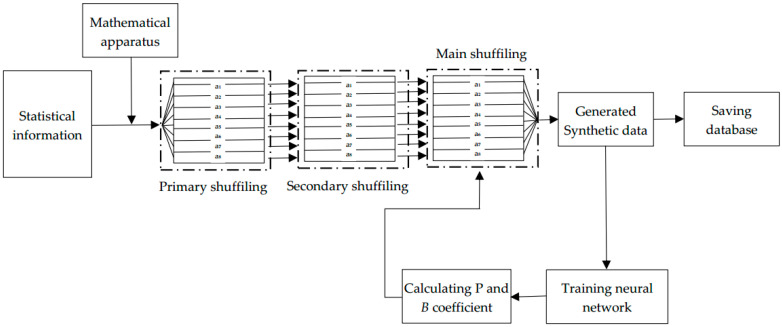
Block diagram of shuffling algorithm.

After studying these papers, we developed a synthetic database based on the statistics presented in this study. The steps for implementing this concept are illustrated graphically in Figure 1.

1. Development of a primary database based on statistical rules and the given statistical data. Based on the selected statistics, the general statistical data were converted into individual patient data. Further details are provided in Section Developing Primary Database.
2. Primary and Secondary shuffling of the data. Usually, the distribution of the data in the initially generated database is highly unbalanced; therefore, it is difficult to bring them to the desired point using the proposed shuffling algorithm during the training process. A primary shuffle should be performed to distribute data uniformly in the database; this method is discussed in detail in Section 4.
3. The database was fed into a loop consisting of the main shuffling and training processes; this step is the main part of our proposed method, in which the primary and secondary shuffled dataset is trained on the neural network. The dataset was shuffled using a special shuffle function and fed again to the neural network depending on the test accuracy value of the neural network; this process was continued until a satisfactory value was obtained for the neural network. The database with the highest performance was selected and saved every time it was tested in the neural network.
4. Evaluation of trained data, and performance enhancement. We will discuss these processes in detail in Section 5.

### Developing Primary Database

We have chosen the case in [28] for this study; this work contains the most detailed information and interconnections of the data. In this paper, the China Cardio Metabolic Disease and Cancer Cohort Study survey was analyzed. This survey was conducted with 93,781 non-diabetic participants nationwide between 2011 and 2016. They examined 14 risk factors for diabetes. These risk factors include education, occupation, unhealthy diet, physical inactivity, current alcohol consumption, current smoking, poor sleep, general or central obesity, insulin resistance, prediabetes, hypertension, and dyslipidemia, gender, age. Age dependence of 13 risk factors was studied in groups, namely (40–55, 55–65, 65–75, and ≥75 years old).

However, we selected 8 factors for this study: age, gender, unhealthy diet, physical inactivity, current alcohol consumption, poor sleep, general or central obesity, and hypertension. We have carefully selected eight factors for our study because they are easily accessible and can be compiled into a comprehensive database. This allows for future verification and comparison, ensuring transparency and credibility. To be more precise, the factors we chose in this work were chosen not because they were the most important, but because they were easy to implement as a proof of concept. That is, the approach does not underestimate the significance of other potentially critical factors, such as insulin resistance and prediabetes. However, just it takes into account that, it is not always possible to find such data of patients, especially data within 5 years. Therefore, if we used all the factors in the article, the possibility of comparing our work with the real database and estimating accuracy would be reduced. Thus we used easily available risk factors. Once a proof of concept has been established using easily available factors, we can expand our database by incorporating more elements in subsequent phases effortlessly, as our method is easy to implement. Moreover, if we want, we can generate a database with more than 14 risk factors, for example, we can add factors such as ethnicity and cardiac disorder. However, for this we should use other survey with more statistical information.

First, the number of patients in each age group was determined. The average age of the patients, standard deviation, and age limits are given for each group. Patient age was calculated using the following formula:

(1) $$F\left(x\right)=\frac{1}{\sqrt{2\pi }}\int_{x_{1}}^{x_{2}}e^{-t^{2}/2}dt$$

where ***x*_1_** and ***x*_2_** are the age boundaries for each group at t-time in the year. We used the following approach to generate the remaining data. The hazard ratios were calculated for each parameter. For incident diabetes associated with risk factors and risk scores according to age group, hazard ratios (HRs) and 95% confidence intervals (Cis) were calculated using Cox proportional hazards models. It is known that the hazard ratio is determined by the following formula:

(2) $$HR=\frac{\left(\frac{O_{d}}{E_{d}}\right)}{\frac{O_{h}}{E_{h}}}$$

where ***O_d_*** is the observed number of events in the group of diabetes, ***O_h_*** is the observed number of events in the group of healthy people, ***E_d_*** is the expected number of events in the group of diabetes, and ***E_h_*** is the expected number of events in the healthy group. Formula (2) was used to arrange the statistical values into groups. It is important to say that, from the beginning, we divided the patients’ data in each age group into 2 subgroups: those who developed diabetes within 5 years and those who did not develop diabetes within 5 years/healthy patients. This study aimed to prevent changes in the statistical values of the dataset. Next, we recorded the values in each subgroup based on Formula (2), using the ratio given in the statistics for diabetic and healthy people.

Although in these statistics, the numbers of men and women were given for each age group, they were adjusted for gender. In other words, the significance of gender has not yet been studied. When we analyzed other studies on the importance of gender, we ascertained that the relationship between diabetes and gender was still under investigation. The risk of developing diabetes among women and men depends on nationality and age [20]. Although it is more common among men in the US, it is also more common among women in East Asian countries. For this reason, we did not use the HR formula to develop the gender data but directly developed the data based on the numbers. As we divided the age group into subgroups and information on the proportion of women and men for subgroups was not given, we also kept the age group proportion within the subgroups.

In addition, it is important to note that all of our data were in the form of zero or one, except for age. Information about the age of the participants was in the range of 40–100. Age was divided by 100 to normalize them to other data.

## 4. Shuffling the Data

### 4.1. Primary and Secondary Shuffling

When the synthetic data were generated based on statistics, it was observed that the data were unevenly distributed. If this database is fed into a loop consisting of a neural network and a special shuffle function, it is possible to develop a database with the desired form. Therefore, primary shuffling was performed before feeding into the neural network. This step includes two shuffling methods: primary and secondary. As the primary shuffling method, we used the Fisher–Yates shuffling method; as the secondary shuffling method, we used a new simple shuffling method. Because the dataset consists mainly of zeroes and ones, shuffling using the Fisher–Yates method does not produce the expected result. Therefore, in addition to the Fisher–Yates shuffle method, we used a secondary shuffling method. This secondary shuffle method operates according to Formulas (3) and (4):

(3) $$a\left[i\right]=a\left[semilength-i\right]$$

(4) $$a\left[semilength\right]=a\left[i\right]$$

### 4.2. Main Shuffling Algorithm

The main shuffling process begins after the primary and secondary shuffling processes. It is worth noting that while the primary and secondary shuffling processes are accomplished only once, the main shuffling process is accomplished in every cycle.

The input data of the function are subgroup data, and their main parameters are the percentage (P parameter) and starting point (B parameter). Based on these parameters, the function shuffles the data within a specific interval of the incoming subgroup database. More precisely, the starting point of the part to be shuffled was determined by parameter B, and its ending point was determined by parameter P. Parameter P determines the percentage of the length of the data to be shuffled. Each time a database is trained and evaluated in a neural network, its value is modified by a special function depending on the accuracy of the network. We initially set these two parameters to 0 and 0.1. This special function is similar to that of the Adam optimizer with slight modifications and is defined by the following formula:

(5) $$v_{t}=\beta_{1}*v_{t-1}-\left(1-\beta_{1}\right)*g_{t}$$

(6) $$s_{t}=\beta_{2}*s_{t-1}-\left(1-\beta_{2}\right)*g_{t}^{2}$$

(7) $$\Delta \omega_{t}=\left|\eta \frac{v_{t}}{\sqrt{s_{t}+\epsilon }}*g_{t}\right|$$

(8) $$\omega_{t+1}=\omega_{t}+\Delta \omega_{t}$$

Here,

η: Initial learning rat
$g_{t}:\mathrm{Gradient}\;\mathrm{at}\;\mathrm{time}\;t\;\mathrm{along}\;\omega^{j}$
$v_{t}:\mathrm{Exponential}\;\mathrm{average}\;\mathrm{of}\;\mathrm{gradients}\;\mathrm{along}\;\omega_{j}$
$s_{t}:\;\mathrm{Exponential}\;\mathrm{average}\;\mathrm{of}\;\mathrm{squares}\;\mathrm{of}\;\mathrm{gradients}\;\mathrm{along}\;\omega_{j}$
$\beta_{1}\beta_{2}:\mathrm{Hyperparameters}$

We gave the following values to the hyperparameters: Initial Learning Rate = 1, $\beta_{1}$ = 0.95, $\beta_{2}$ = 0.99, ϵ= **0.0001**. The coefficient ***B*** is increased by 0.12 after each cycle. If the value of coefficient ***B*** exceeds 1, the operation B=B−1 will be performed. If the endpoint exceeds the length of the array, the value of coefficient ***B*** is set to zero.

After the start and end points of the shuffling were determined, the numbers in between were shuffled using the main shuffle function. Let the data be expressed in the form of matrix ***A*** where $A=\left|\begin{array}{cccc} a_{11} & a_{21} & \ldots & a_{81}\\ a_{12} & a_{22} & \ldots & a_{81}\\ \ldots & \ldots & \ldots & \ldots \\ a_{1i} & a_{2i} & \ldots & a_{8i} \end{array}\right|$. All elements of the array, except for the first, were 0 or 1. The last row shows the diagnostic values; therefore, the main shuffle function is only performed on columns 2–7 for each column separately. The values in the first column were shuffled according to the French–Yates algorithm in a given interval. The main shuffling function is defined as follows:

(9) $$a_{out}\left(j,k\right)=\left|\begin{array}{c} 1\\ 1\\ \ldots \\ 1 \end{array}\right|-\left|\begin{array}{c} a_{j1}\\ a_{j1}\\ \ldots \\ a_{jk} \end{array}\right|$$

Here, $a_{j,k}$—is the ***j***th column of the subgroup data obtained in the given interval. This function converts 0 s to 1 s and 1 s to 0 sin in the given interval. However, the statistical distribution of the database was distorted. To avoid this, the number of 1 s in a given interval is compared with the previous interval as follows:

(10) $$\Delta a\left(j,m\right)=\sum_{m=0}^{k}a_{0}\left(j,m\right)-\sum_{m=0}^{k}a_{out}\left(j,m\right)$$

$$N=\left|\Delta a\left(j,m\right)\right|$$

If $\Delta a\left(j,m\right)$ is greater than 0, this means there are ***N*** more 0 s converted to 1 s than 1 s converted to 0 s. Therefore, the next interval is selected from the endpoint to the end of the array, and the ***N*** interval within this interval is converted to zero. If $\Delta a\left(j,m\right)$ is less than zero, the reverse operation will be carried out, that is, ***N*** zeroes within this interval are converted to one. Therefore, the statistical distribution of the database was preserved.

### 4.3. Training for Loop

The database contained information on two categories of patients: 87,610 patients were followed up and remained healthy; 6171 patients developed diabetes during this time. As shown, the dataset was highly unbalanced. Typically, neural networks are likely to overfit when trained using such databases. Therefore, we multiplied the data for a small number of classes several times. Considering that the data are not real and unique and will be shuffled several times in the next step, we simply added the same data several times. After increasing the data, the number of people with diabetes was 84,692. Subsequently, the data were used for the next step. It is worth mentioning once again that the data that passed through the first step were not in the form of a single dataset but in the form of separate subgroups. The number of data points in these subgroups is presented in Table 1.

The second step consists of two processes: shuffling the data and training the neural network on the prepared data. A special main function is used for shuffling.

After shuffling, the subgroup data were combined into a single database and divided by a ratio of 8:2 into training and test databases. The training dataset was then transferred to the NN, which comprised 16, 64, and 32 consecutive hidden layers. After each linear layer, a ReLU activation layer was formed. The neural network was trained over five epochs and evaluated using the test dataset. These two processes (shuffling and training) were repeated until the desired results were achieved.

## 5. Results and Discussion

**Figure 2:**
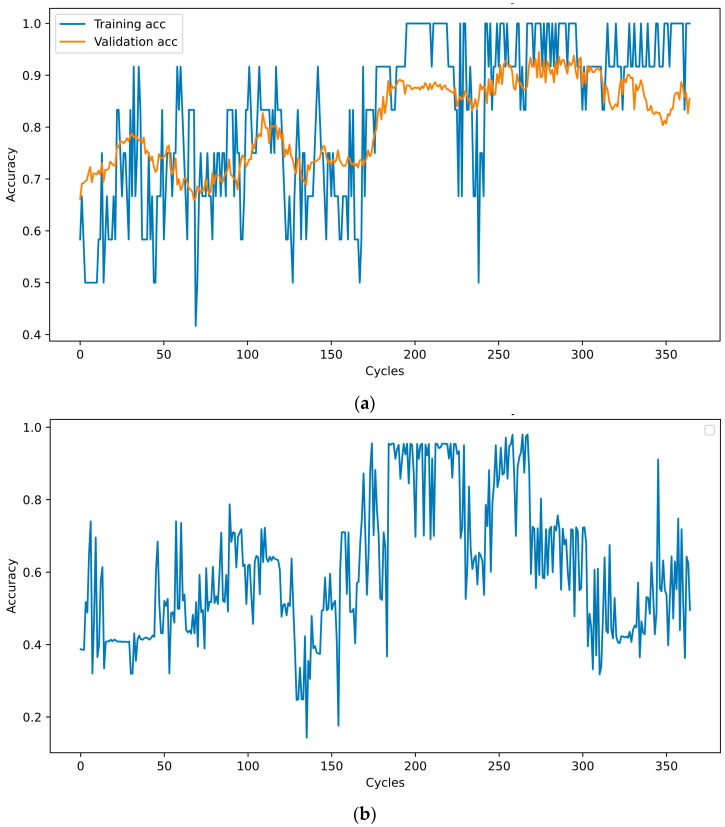
Training, validation (**a**) and test (**b**) accuracy at the end of each training cycle.

In each cycle, the neural network was trained for five epochs, and its performance was assessed using a test database. The test accuracy was sent to the optimizer to determine the percentage and starting points. When the new values of the percentage and starting point are determined, the cycle starts again, and the data are reshuffled and transmitted to the NN. This cycle was repeated 100 times. The training, validation, and test accuracies at the end of each cycle are shown in Figure 2. A database was saved after each cycle. Among these, the one with the highest number of test results was selected. When training NN with this dataset, the training accuracy was 100%, and the test accuracy was 94.4%.

**Figure 3:**
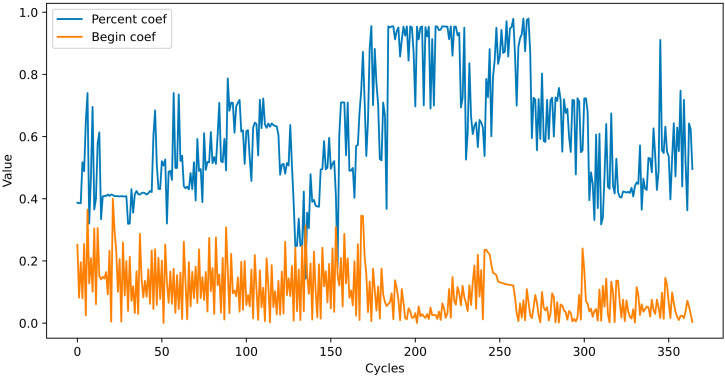
Values of P and B coefficients at the end of each cycle.

As mentioned above, at the end of each cycle, the P- and B-coefficient values change according to (7). Their values over 100 cycles are shown in Figure 3.

### 5.1. Evaluation of the Method

Various methods have been proposed to evaluate synthetic databases. However, because the proposed method is completely synthetic, these evaluation methods are unsuitable for evaluating our method. The following methods were used to evaluate the method.

**Figure 4:**
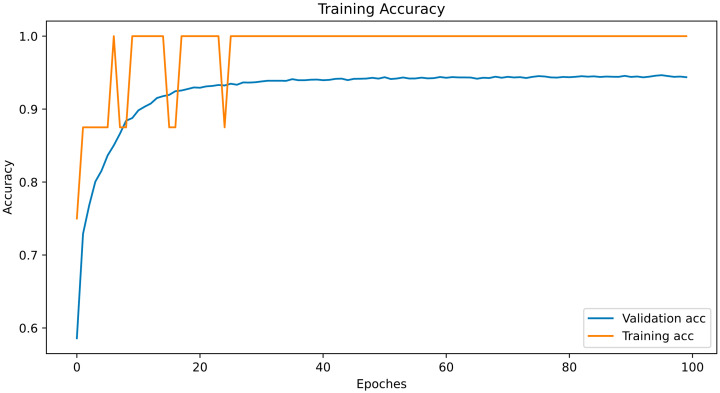
Training and validation accuracy of the neural network trained on the generated dataset.

First, the database that exhibited the best results when the neural network that was trained was selected. Subsequently, the selected database was trained in a completely new neural network to evaluate the real value of the dataset, as this dataset was intended to train the AI algorithms. The network architecture is presented in Table 2, and the training process is illustrated in Figure 4. The database was split randomly with random state 46, in an 8:2 portion, into the training and test sets.

As shown in Figure 3, the test accuracy reached 90% at the 10th epoch; while at the 20th, it reached 94%; and after that, it remained approximately the same. We extracted the dataset that showed the highest accuracy (94.4%) in these cycles and uploaded it to Kaggle for public use.

**Figure 5:**
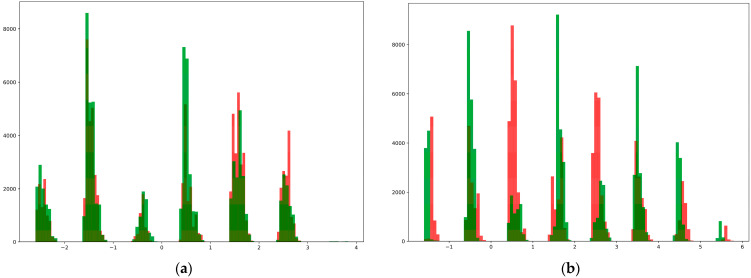
Data distribution in raw dataset (**a**) and generated dataset (**b**).

Second, they were plotted in the form of a histogram to determine the distribution of the data in the database. For this purpose, we divided the data into two groups: those who developed diabetes within five years and those who remained healthy. We then obtained two matrices of the forms (84,692, 8) and (87,598, 8). The first seven columns of these matrices show the disease risk factors and the last column shows the diagnosis. We initially added seven columns, separated the resulting columns by values, and represented them in the form of 100 histograms (Figure 4). For a comparative study of the data distribution, histograms of the dataset before shuffling and the dataset with the highest results during shuffling are shown in Figure 5.

As can be seen from the above, the data of different classes in the synthetic dataset generated by the proposed method were well distinguished from each other, and because of this, it was possible to achieve high accuracy when using them to train the neural network.

### 5.2. Discussion

In this study, we propose a method for generating a synthetic dataset without complex mathematical operations or an initial database. This technique is superior to the previously proposed strategies in numerous respects.

Firstly, most works [22,23] that generated a fully synthetic database calculated the desired statistical distribution from the real database and then used it to generate synthetic data. However, it is important to note that using real datasets in research can be challenging due to privacy concerns and legal restrictions. Accessing and utilizing such datasets may require permissions and agreements that are not always easy to obtain. Therefore, having a method that does not rely on real datasets would greatly simplify the process and make it more accessible for researchers [29,30,31,32].

While our proposed method utilizes only the given statistics of the desired database, by using the proposed method with these given statistics, we can generate synthetic data that closely resembles the original dataset without compromising privacy or legal constraints.

Moreover, this approach allows for greater control over the generated data. Researchers can manipulate and experiment with different scenarios by adjusting the statistical parameters provided. This flexibility enables them to explore various possibilities and test hypotheses without being limited by an existing dataset.

Secondly, although the work in [24] has several advantages and is a highly reliable method, it uses a complicated method. In order to create the dataset, medical conclusions and guidelines were used. While this ensures accuracy and credibility, extracting the correct and necessary medical instructions and rules can be both time consuming and expensive. One of the main challenges in using medical conclusions and guidelines is their sheer volume. The vast amount of information available makes it difficult to sift through and extract only what is relevant for creating the dataset. This requires extensive research and analysis, which can be a time-consuming process.

Moreover, obtaining accurate medical instructions and rules often involves consulting experts in the field. These experts may charge high fees for their services, making it expensive to gather the necessary information for creating the dataset. Additionally, medical knowledge is constantly evolving with new research findings and updated guidelines being published regularly. This means that maintaining an up-to-date dataset requires continuous effort and investment.

However, our proposed method offers the possibility of generating databases in a semi-automatic way with minimal human intervention, and most importantly, without the involvement of medical professionals. Thus, it can significantly reduce the time and effort required. Moreover, eliminating the involvement of medical professionals further streamlines the process. Additionally, cost plays a crucial role in any project implementation. Traditional methods involving medical professionals can be expensive due to their expertise and time commitment. However, with our semi-automated approach, costs are significantly reduced as there is no need for specialized personnel or extensive training. Furthermore, speed is a critical factor in today’s fast-paced world. Our proposed method ensures the rapid generation of databases. This enables researchers and programmers to access up-to-date databases promptly for analysis and decision-making purposes.

Another advantage is that, in contrast to [25], we used a rule-based shuffle method instead of a random shuffle method to achieve this goal; this helped us achieve our goals faster.

Now, if we turn to the issue of evaluating the quality of the developed method, it is known that today various methods have been developed for the evaluation of synthetic data. However, some are designed to evaluate synthetic images [33], others to determine the level of security [34], and others to evaluate the difference between the distributions of synthetic and real images [35]. However, none of the above methods were suitable for evaluating the proposed method. As we do not have a real database, there is no privacy issue, and simultaneously, there is no possibility of comparing the statistical distribution with that of the real dataset. In such cases, certain authors have suggested the use of specific evaluation methods. For example, a unique evaluation method was used in the work [24], and some statistical data in the database were compared with those of other real statistical information. Similarly, we used a unique approach to evaluate the results of our study. Our goal was to develop a database for training disease classification and prediction algorithms with two main goals. The first was to preserve the statistical distribution of the survey used to construct the dataset, and the second was to ensure that the synthetic data belonging to two different classes were maximally different from each other. In our method, actions at all stages of the proposed method assume the preservation of the statistical distribution of the survey; that is, the generated synthetic dataset is identical to the statistical data of the survey. We expressed the data distribution as a histogram to evaluate the dissimilarity between different classes. As shown in Figure 5, the data in the created database are satisfactorily separated.

One major limitation of the proposed method is relying solely on one survey. Different surveys often focus on different aspects or variables related to a particular topic. Combining these various perspectives allows for a more comprehensive analysis and provides researchers with a broader understanding of the subject matter. Additionally, incorporating data from multiple surveys enhances the generalizability of findings. It helps in identifying patterns and trends across diverse populations or contexts. This broader scope strengthens the validity and reliability of the database. To address this limitation, future research should aim to modify this method to integrate data from multiple surveys seamlessly.

## 6. Future Work

Currently, much work is being conducted to extract medically important characteristics from existing datasets [36], the main goal of which is to define and evaluate the main risk factors that cause the disease and to use them in disease prediction or diagnosis. By contrast, our proposed method aims to generate a dataset based on given risk factors. In the future, by analyzing the dependence of risk factors and information in the dataset using these two methods, it will be possible to develop an algorithm that determines the relationship between them, which will be an important tool for diagnosis [37,38,39,40,41,42,43].

## 7. Conclusions

In this study, with the help of a special shuffle operator, a synthetic dataset was generated that fully represented the statistical data of the survey conducted by [44] over five years. This database contains two classes: data on patients who developed type 2 diabetes and data on those who remained healthy during a 5-year follow-up. This generated dataset can be used to train AI algorithms designed to predict type 2 diabetes five years in advance. To assess the suitability of the database for this purpose, a neural network was trained using this dataset, and a test accuracy of 94.4% was achieved. From the above, it can be concluded that the accuracy, reliability, and simplicity of the proposed method are important.

While relying on one survey is the limitation of the method, considering information from different surveys is crucial. Future research should focus on creating methods that can encounter several research/survey papers’ information to enhance accuracy, comprehensiveness, generalizability, and reliability of the generated database. In conclusion, the proposed easy and semi-automotive method offers a solution by utilizing a neural network and special shuffling function. This approach not only reduces the difficulty associated with generating synthetic medical data but also provides satisfactory results in a more efficient manner.

## Figures and Tables

**Table 1:** Information about subgroup data.

Age Group, Years	40 to <55		55 to <65		65 to <75		≥75	
Subgroup	Did not develop diabetes	Developed diabetes	Did not develop diabetes	Developed diabetes	Did not develop diabetes	Developed diabetes	Did not develop diabetes	Developed diabetes
Number of patients	42,825	2306	31,355	2478	11,570	1176	1851	203
Number of male patients	12,505	673	10,786	852	4766	484	795	87
Number of female patients	30,320	1633	20,569	1626	6804	692	1056	116

**Table 2:** Layers parameters of neural network.

#	Layer Name	Properties
**1**	Input layer	8 nodes
**2**	Hidden layer	16 nodes
**3**	Dropout layer	0.2 coefficient
**4**	ReLu	
**5**	Hidden layer	64 nodes
**6**	Dropout layer	0.2 coefficient
**7**	ReLu	
**8**	Hidden layer	32 nodes
**9**	Dropout layer	0.2 coefficient
**10**	ReLu	
**11**	Hidden layer	16 nodes
**12**	ReLu	
**13**	Hidden layer	1 nodes
**14**	Output (Sigmoid) layer	

## Data Availability

Not applicable.
